# Review of the use of prophylactic drain tubes post‐robotic radical prostatectomy: Dogma or decent practice?

**DOI:** 10.1002/bco2.20

**Published:** 2020-06-09

**Authors:** Tatenda C. Nzenza, Simeon Ngweso, Renu Eapen, Nieroshan Rajarubendra, Damien Bolton, Declan Murphy, Nathan Lawrentschuk

**Affiliations:** ^1^ Department of Surgery Austin Hospital University of Melbourne Melbourne VIC Australia; ^2^ Young Urology Researchers Organisation (YURO) Melbourne VIC Australia; ^3^ Department of Surgical Oncology Peter MacCallum Cancer Centre Melbourne VIC Australia; ^4^ Department of Urology Fiona Stanley Hospital Murdoch WA Australia; ^5^ Olivia Newton‐John Cancer Research Institute Austin Hospital Melbourne VIC Australia; ^6^ EJ Whitten Prostate Cancer Research Centre Epworth Healthcare Melbourne VIC Australia

**Keywords:** drainage, drain tube, prostatectomy, RARP

## Abstract

**Objective:**

To assess the necessity of routine prophylactic drain tube use following robot‐assisted radical prostatectomy (RARP).

**Method:**

We performed a literature review using the Medline, Scopus, and Web of Science databases with no restriction of language from January 1900 to January 2020. The following terms we used in the literature search: prostatectomy, radical prostatectomy, robot assisted, drainage, and drain tube.

**Results:**

We identified six studies that examined the use of routine prophylactic drain tubes following RARP. One of these studies was a randomized study that included 189 patients, with 97 in the pelvic drain (PD) arm and 92 in the no pelvic drain (ND) arm. This non‐inferiority showed an early (90‐day) complication rate of 17.4% in the ND arm versus 26.8% in the PD arm (*P* < .001). Another non‐inferiority randomized control trial (RCT) showed a complication rate of 28.9% in the PD group versus 20.4% in the ND group (*P* = .254). Similarly, the other studies found no benefit of routine use of prophylactic drain tube after RARP.

**Conclusion:**

Drain tubes play a role during robotic‐assisted radical prostatectomy, however, following a review of the current available literature, they can be safely omitted and we suggest that clinicians may be selective in their use.

## 1 INTRODUCTION

The historical use of surgical drains can possibly be attributed to the classical Greek physician Hippocrates who used hollow tubes to treat ascites.^1^ Currently deployed for a number of indications, insertion of surgical drains has remained a standard feature of abdominopelvic surgery. Ongoing contention exists, however, regarding routine insertion of abdominopelvic surgical drains in many situations.

Debate regarding insertion of surgical drains dates back to the turn of the century with Lawson Tait, pioneering abdominopelvic, and gynecological surgeon stating, “when in doubt, drain”.^2^, ^3^ In stark contrast, William Halstead, a similarly influential 19th century surgeon and founding professor at Johns Hopkins Hospital famously stated, “no drainage at all is better than the ignorant employment of it”.^2^, ^3^ Contention surrounding insertion of surgical drains during abdominopelvic surgery has arisen due to a scarcity of evidence supporting their use, particularly when employed for purposes of prophylactic drainage. Similarly, there are recognized risks associated with insertion of pelvic drains including pain, infection, and prolonged hospital stay.^4^

Robotic‐assisted radical prostatectomy (RARP) is an internationally accepted standard approach for the management of localized prostate cancer.^5^ With the rapid uptake of RARP internationally, the question as to the necessity for routine insertion of surgical drains during this particular modality of procedure remains unanswered.^5^ The rationale for insertion of a pelvic, surgical drain following RARP is multifaceted, with many indications established historically not necessarily retaining relevance within the context of robotic surgery. For example, the running anastomosis made possible with the robotic approach is more watertight than the interrupted suture technique utilized with an open or laparoscopic prostatectomy, making anastomotic urine leak, and subsequent urinoma less common.^6^

The primary purpose of the following review was to examine the current body of literature pertaining to the insertion of routine pelvic surgical drains during RARP. We aimed to determine whether routine insertion of pelvic surgical drains is necessary following RARP.

## 2 METHOD

A literature review was performed using Medline, Scopus, and Web of Science to identify relevant articles published up until January 2020. The following terms were used to identify relevant articles “prostatectomy,” “radical prostatectomy,” “robot assisted,” “drainage,” and “drain tube.” There were no restrictions placed on language, year, or study design. About 126 articles were imported into Endnote x9 where duplicates, abstracts, and irrelevant titles were filtered out. For analysis, we included full text publications that compared outcomes of RARP with versus without pelvic drain tube.

Inclusion criteria:

Original studies
Robotic prostatectomy
Comparative studies (drain vs no drain) and this includes randomized as well as non‐randomized studies
Report on postoperative complication rates

Exclusion criteria:

Abstracts
No comparison arm
Animal studies

## 3 RESULTS

We identified six studies that examined the use of prophylactic drain tubes following RARP with combined total of 8338 cases analyzed. One randomized study by Chenam et al^5^ was identified which included 189 patients, with 97 in the pelvic drain (PD) arm and 92 in the no pelvic drain (ND) arm. The early (90‐day) complication rate was 17.4% in the ND arm vs 26.8% in the PD arm (*P* < .001). The rates of symptomatic lymphocoele were 2.2% in the ND arm compared to 4.1% in the PD arm (*P* = .7). Another randomized, non‐inferiority study by Porcaro et al^7^ involving 112 patients (two excluded from analysis) showed an overall complication rate of 28.9% in the PD group versus 20.4% in the ND group (*P* = .254). There were no complications classed as Clavien 3 or above in the ND group compared to three cases in the PD group. A large multicenter retrospective analysis by Kirmiz et al^8^ involving 6746 RARP cases (66% had drain) between 2014 and 2017 reported more complications in the PD arm (17.7%) compared to the ND arm (11.1%). Pelvic lymph node dissection (PLND) was carried out in 77.5% of the cases and uniformly between the two study groups. This analysis involved surgeons who routinely placed drain tubes and those who selectively placed drain tubes after RARP. The authors attempted to minimize the effects of selection bias by analyzing the outcomes based on the surgeon patterns of drain tube use (routine vs selective) and apart from increased length of stay in the routine drain tube cohort, there were no differences between the two groups. Messer et al^9^ describes results of 651 patients from a single surgeon comparing two periods; one period using PD, and then, another period with ND. The frequency of complications (Clavien 2‐5) was 7% in the ND cohort compared to 8.7% in the PD cohort. Danuser et al^10^ prospectively analyzed the necessity of a pelvic drain following extended pelvic lymph node dissection (ePLND) with open radical retropubic prostatectomy (RRP) or RARP. There were 331 patients spread across four groups with group 4 (126 patients) including all the RARP patients, all of which had no drain. The incidence of lymphocoeles in the RARP group was 3% and only 1% developed symptomatic lymphocoeles. In another study, Sharma et al^11^ reported results of 325 patients undergoing open RRP and RARP with PD or ND. They reported complication rate of 6% in the ND arm and 11% in the PD arm (Table 1).

**Table 1:** Summary of studies comparing drain tube vs no drain tube post‐robotic prostatectomy

Year	Author	Study design	Surgical technique	Cases (DT vs no DT)	Complications			Country
					Drain	No drain	*P*‐value	
2019	Kirmiz et al^8^	Retrospective	RARP	6746 (4451 vs 2295)	17.7%	11.1%	<.0001	USA
2018	Porcaro et al^7^	RCT	RARP	110 (56 vs 54)	20.4%	28.9%	.254	Italy
2018	Chenam et al^5^	RCT	RARP	189 (97 vs 92)	26.8%	17.4%	<.0001	USA
2014	Musser et al^9^	Retrospective	RARP	637 (379 vs 258)	8.7%	7%	–	USA
2013	Danuser et al^10^	Prospective	ORP/RARP	331 (126 RARP)	–	3% RARP	–	Switzerland
2007	Sharma et al^11^	Prospective	ORP/RARP	325 (70 vs 255)	11%	6%	>.05	USA

## 4 DISCUSSION

Drain tubes are used following abdominal surgery to serve as an early indicator of an anastomotic leak, to decrease the accumulation of collections (e.g., blood, pus, or infected fluids) or to prevent the accumulation of air (dead space). A common reason, however, for the use of drain tubes is “surgeon's choice” usually based on habit or personal experience. Despite the usefulness of drain tubes in surgery, they are not without complications. An often debated complication of drain tubes is their role in increasing the risk of infection including surgical site infections.^3^ DTs can also be associated with increased postoperative pain, delaying hospital discharge, and limiting patient mobility.^4^ DT’s can sometimes induce an anastomotic leak due to mechanical, negative pressure, or suction effect when the DT is overlying the anastomosis.^12^ DT’s have also been reported to be retained within body cavities and a second procedure to remove the DT or retrieve a foreign body is sometimes necessary.^13^, ^14^ Another recognized disadvantage of DT’s pertains to a lack of clarity regarding the appropriate type of DT to be employed in particular situations and the duration of drainage required.

Multiple operation specific studies have been conducted in an attempt to reach a consensus regarding the use of abdominopelvic surgical drains. A meta‐analysis which included 11 randomized control studies looked at the role of prophylactic drainage in reducing complications related to colorectal anastomosis. About 1803 patients were included in this analysis which found no statistically significant difference between the rates of complications in the no drain group when compared to the drain group. The authors concluded that the routine use of prophylactic drain tubes following colorectal anastomosis did not have a benefit in the reduction of complications.^15^ Drainage postgastrectomy has also been analyzed in a meta‐analysis which included four randomized control trials (438 patients).^16^ Similarly, the authors did not find any convincing evidence to support the routine use of prophylactic drainage after gastrectomy. For pancreatic surgery, a meta‐analysis comprised of five randomized control trials and eight non‐randomized studies failed to reach a clear conclusion on whether there was a benefit of routine use of prophylactic drainage.^17^

In Urological surgery, the role for the routine use of prophylactic drainage is also being explored. A randomized study comprised of 106 patients undergoing open nephrectomy found the presence of a surgical drain tube to not affect the rate of complications (*P* = .249).^18^ Despite the randomized nature of the study, we should however acknowledge that these results were based on a small sample size. In a series of 208 patients undergoing laparoscopic radical prostatectomy with a running urethrovesical anastomosis (RUVA), the authors concluded that routine use a prophylactic drain was not necessary.^19^ The need for a routine prophylactic drain was also shown to be unnecessary in a series of 552 patients undergoing RRP given there were no concerns with the anastomosis.^20^ Similarly, in their series of 116 patients, Savoie et al concluded that the use of routine prophylactic drain could be avoided following RRP.^21^

With regards to the routine use of prophylactic drains following RARP, the results presented in this analysis indicate that we can omit drains in select cases. However, only two of the studies were randomized, and these studies also had limitations. One study did not accrue as initially intended and as such the final sample study in this analysis is small.^5^ The other RCT it was a single center study with small numbers and only two surgeons.^7^ This raises questions about applicability of the findings to the general population. The other studies are largely retrospective or single surgeon series which may, therefore, have inherent bias.^8^, ^9^, ^10^, ^11^ Despite the noted study limitations, these results still indicate a role of selective use of surgical drain tubes based on factors including specific patient characteristics, concerns with the anastomosis or issues with hemostasis rather than adopting a blanket rule for all patients where the default action is to place a drain tube. The concept of omitting a drain tube would also be in‐line with enhanced recovery after surgery (ERAS) protocols and the growing notion of same‐day discharge RARP which is getting explored more and more in some parts of the world.^22^, ^23^

A potential concern following prostatectomy is the development of a symptomatic lymphocoele. However, after RARP with lymph node dissection, symptomatic lymphocoeles are particularly rare. Keskin et al reported an incidence of symptomatic lymphocoeles of 2.5% in their series of 521 patients who underwent RARP with Eplnd.^24^ The rate of symptomatic lymphocoele after RARP with PLND is much lower compared to the reported incidence following open PLND.^25^ Reasons for this are debatable with some studies suggesting the rate of lymphocoele formation as being associated with the extent of lymph node dissection but similarly, certain studies have also found no association between lymphocoele development and extent of lymph node dissection.^26^, ^27^, ^28^ Prophylactic drain tube placement may not prevent lymphocoele formation as indicated by the results presented in this review. As more studies explore this issue, a drain tube score could potentially be established based on more reliable evidence to help guide clinicians in assessing the need for a drain on an individualized, case‐specific basis, and determined by specific patient, disease, and surgical factors.

## 5 CONCLUSION

Drain tubes play a role during robotic‐assisted radical prostatectomy, however, following a review of the current available literature, they can be safely omitted, and thus, we suggest that clinicians should be selective when it come to their use rather than preemptive.

## CONFLICTS OF INTEREST

The authors declare that there is no conflict of interests regarding the publication of this paper.
